# Nucleoporin insufficiency disrupts a pluripotent regulatory circuit in a pro-arrhythmogenic stem cell line

**DOI:** 10.1038/s41598-019-49147-4

**Published:** 2019-09-03

**Authors:** Claudia C. Preston, Emily C. Storm, Ryan D. Burdine, Tyler A. Bradley, Andrew D. Uttecht, Randolph S. Faustino

**Affiliations:** 1 Genetics and Genomics Group, Sanford Research, 2301 E. 60th Street N., Sioux Falls, SD 57104 USA; 2 Department of Pediatrics, Sanford School of Medicine of the University of South Dakota, 1400 W. 22nd Street, Sioux Falls, SD 57105 USA

**Keywords:** Embryonic stem cells, Stem-cell research

## Abstract

Nucleoporins have been reported to regulate pluripotent biology, but how they do so remains partially characterized. This study examined the effects of *nup155* gene disruption on mouse embryonic stem cells to gain insights into possible mechanisms by which nucleoporins regulate pluripotency in a pro-arrhythmogenic stem cell line. Embryonic stem cells with gene-trapped *nup155* exhibited aberrant colony morphology underscored by abnormal transcriptome remodeling. Bioinformatic analysis of whole transcriptome data from *nup155*^+/−^ embryonic stem cells revealed changes in a variety of non-coding RNA elements, with significant under expression of *miR291a*, *miR291b*, *miR293*, and *miR294*. These miRNAs are members of the larger regulatory *miR290–295* cluster that regulates pluripotency and are controlled by the canonical stem cell-related factors SOX2, OCT4, and NANOG. Expression analysis of these factors revealed downregulation in all three, supported by biochemical profiling and image analysis. These data implicate disruption of the *miR*-SOX2/OCT4/NANOG regulatory circuit occurs downstream of *nup155* gene lesion.

## Introduction

Nucleoporins (nups) are a family of highly conserved proteins that comprise the nuclear pore complex (NPC), an intricate macromolecular structure that spans both layers of the nuclear membrane and facilitates bidirectional exchange between cytoplasmic and nuclear compartments^1,2^. The NPC plays an essential role in regulating gene expression by controlling nucleocytoplasmic transport of proteins and mRNA^3^, and mounting evidence implicates significant roles for nups in normal development as well as pathogenic processes^4,5^. This is supported by multiple studies that report nup mutations and expression deficiencies associated with cardiac, neurogenic, and reproductive disorders, as well as with various forms of cancers^6^. For example, previous work using a murine embryonic stem cell (ESC) line containing a heterozygous truncation of the NUP155 gene (*nup155*^+/−^) associated with atrial fibrillation (AF) revealed that NUP155 disruption impaired nuclear transport of HSP70^7^. This expression dysfunction was suggested as one of the underlying causes of arrhythmogenesis-associated sudden cardiac death in patients expressing a homozygous *NUP155-R391H* mutation^7^. Building on this observation, independent and unbiased bioinformatic gene expression analysis of transcriptome dynamics in NUP155*-*compromised ESCs by Preston *et al*. revealed that system wide transcriptome remodeling had occurred in the mutant line, with re-prioritized cellular functions and reorganized gene regulatory networks^8^. Furthermore, embryoid body-derived differentiation of these ESCs into beating cardiac foci recapitulated an arrhythmogenic phenotype that demonstrated impaired reponses to agonist treatment^8^.

Given the functional impacts of NUP155 in RNA regulation and gene network biology^8–11^, we investigated how disruption of the *Nup155* allele affected the non-coding transcriptome in ESCs. Our results revealed decreased expression of pluripotency factors, as well as alterations in non-coding RNA (ncRNA) species that affected ESC biology. This is significant in light of observations that gene regulatory functions and associated disease phenotypes are recognized for a variery of NUPs with mutations in NUP155 linked to arrhythmogenesis^7,12^. Our present work reports that non-coding transcriptome remodeling occurs in NUP155 insufficient pluripotent stem cells and suggests that phenotype impairment related to NUP155 deficiency may happen well in advance of cardiac manifestation.

## Results

### ESC colony disruption in insufficient *nup155*^+/−^ cell lines is associated with underlying transcriptome remodeling

**Figure 1:**
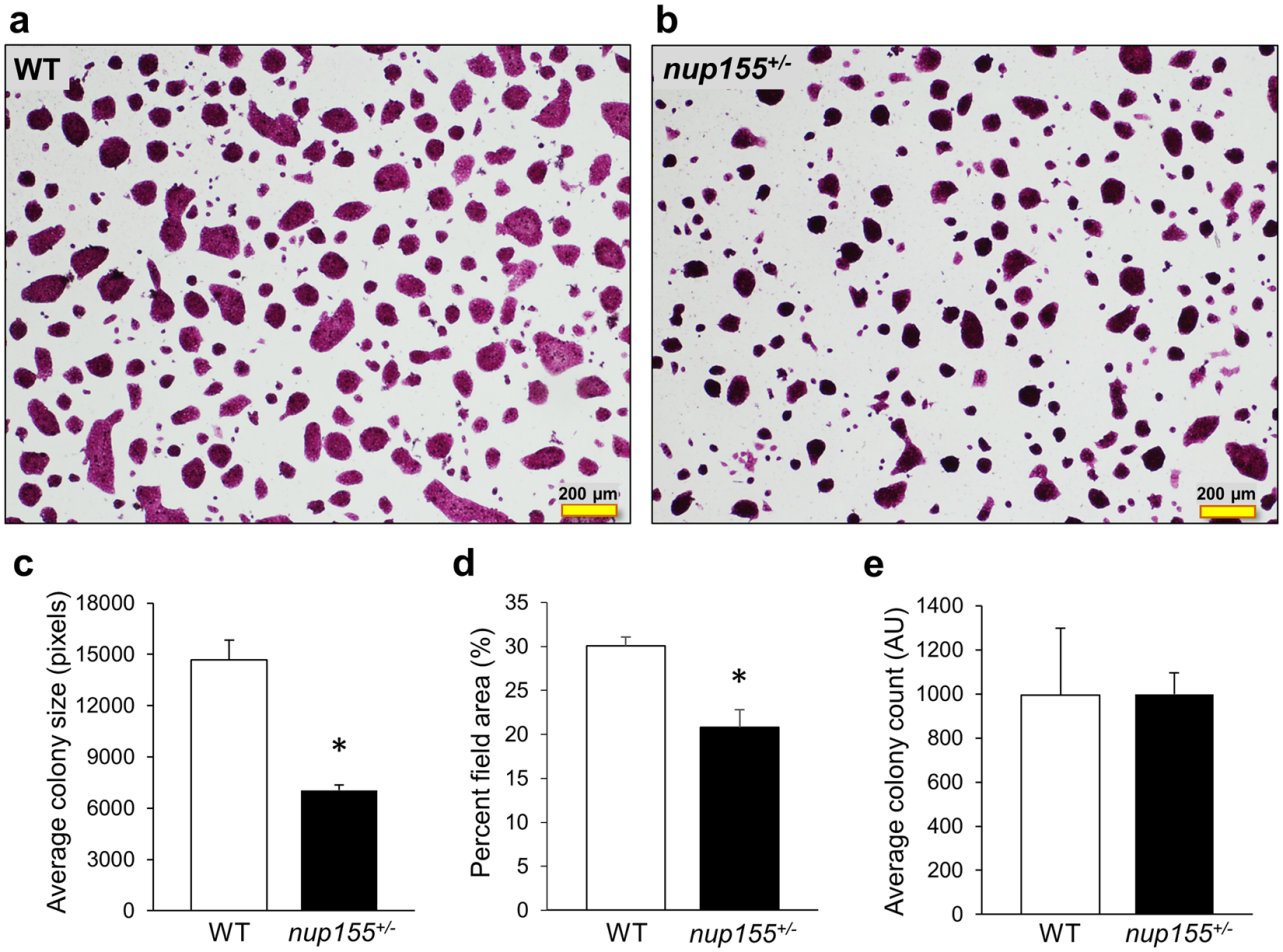
Nucleoporin insufficiency blunts ESC colony mass. (**a**,**b**) Representative images of alkaline phosphatase staining in mouse ESC colonies in WT (left panel) and *nup155*^+/−^ (right panel) conditions showing positive stain in both cell lines. (**c**) Image analysis revealed significant reduction in size of *nup155*^+/−^ ESC colonies (52%, p < 0.05) as well as (**d**) lower percent area of colonies per field compared to WT (21% vs 30% respectively, p < 0.05). (**e**) No change was observed in overall colony number between conditions (p = 0.99).

ESC colonies from *nup155*^+/−^ and wild type (WT) conditions depicted positive alkaline phosphatase test (Fig. 1a,b). Image analysis of insufficient NUP155 ESCs exhibited a significant smaller colony size (n = 4, p < 0.05, Fig. 1c) and overall diminished colony coverage (p < 0.05, Fig. 1d) than WT counterparts (n = 4), despite similar total colony counts (Fig. 1e). This phenotype impairment is supported by decreased protein expression of NUP155 (Supplementary Fig. S1) and diminished proliferation seen in NUP155 deficient ESC line (Supplementary Fig. S2), which is in line with compromised differentiation linked to changes in the underlying coding transcriptome^8^. To further investigate potential systems biology changes driving NUP155-deficient phenotypes, we focused on the non-coding transcriptome potentially impacted by NUP155 insufficiency.

### Non-coding transcriptome remodeling occurs in *nup155*^+/−^ pluripotent stem cells

**Figure 2:**
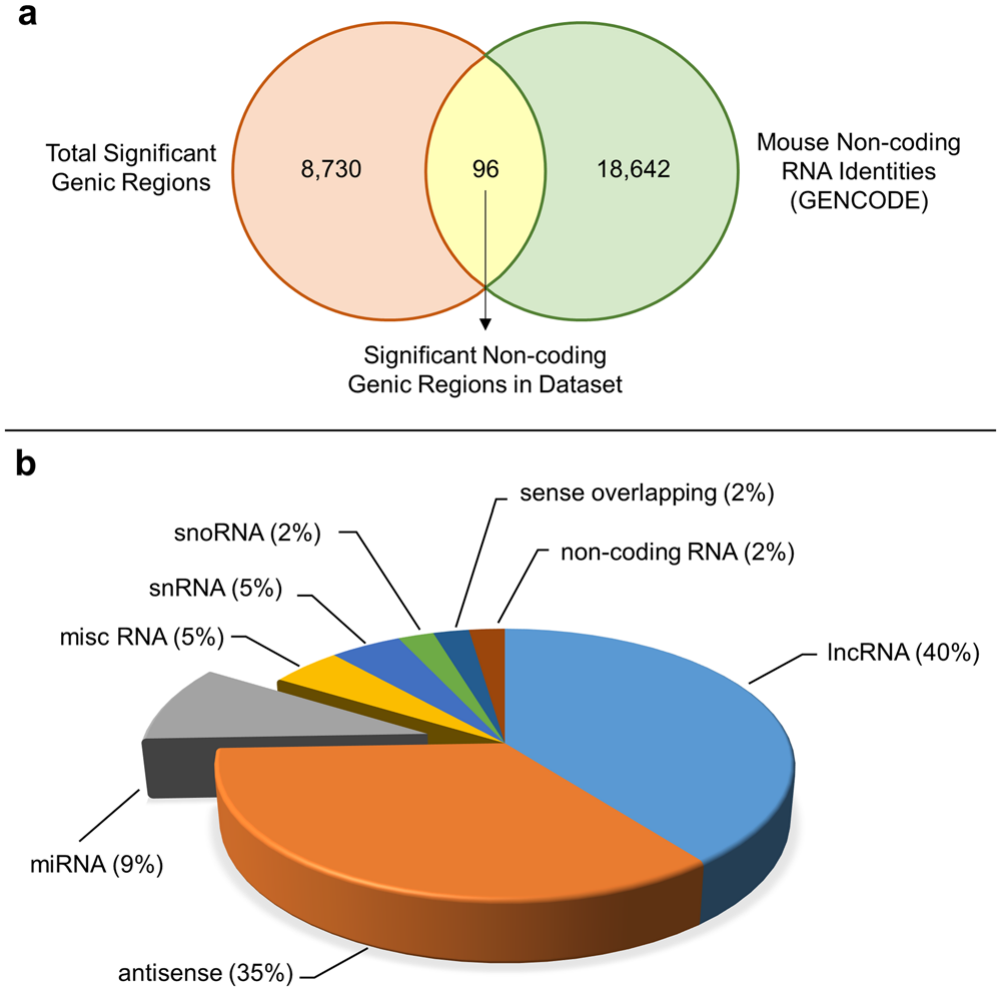
Remodeling of the non-coding sub-transcriptome in *nup155*^+/−^ ES cells. (**a**) Venn diagram intersecting significantly changing genic regions from GSE111596 dataset (8,822; light orange) with all known non-coding RNA (ncRNA) entries from the mouse GENCODE database (18,734; light blue). From this, 96 distinct ncRNAs were unique to the present study (light yellow). (**b**) Transcriptome deconvolution revealed that the majority of ncRNAs detected in our analysis were long non-coding RNAs, antisense RNAs and microRNAs, with the remainder belonging to other RNA species (see text). Shown is the categorical breakdown for down regulated ncRNAs enriched following analysis (43 entities, fold change >2, p < 0.05). Abbreviations – lncRNA, long non-coding RNA; misc, miscellaneous; miRNA, microRNA; rRNA, ribosomal RNA; snoRNA, small nucleolar RNA; snRNA, small nuclear RNA.

The underlying transcriptome of a NUP155 deficient ESC line is significantly remodeled^8^. To investigate the possibility that the ncRNA subtranscriptome was altered, our previous dataset (GSE111596) was analyzed to identify distinct genic regions including coding and non-coding regions. These were cross referenced with mouse GENCODE identifiers to prioritize a total of 96 ncRNA transcripts significantly changing in our samples (n = 5 each condition, p < 0.05, fold change >2, Fig. 2a). These were divided into up and downregulated categories, of which there was a similar distribution of ncRNAs (Fig. 2b, Supplementary Fig. S3). We found 43 significantly downregulated and 53 upregulated ncRNAs sub categorized as: long non-coding RNAs (lncRNAs), antisense RNAs, miRNAs, small nucleolar RNAs (snoRNAs), small nuclear (snRNAs), ribosomal RNAs (rRNA), sense overlapping transcripts, miscellaneous or unknown non-coding RNAs (Supplementary Table S1).

### Expression of a downregulated miRNA cluster associated with *nup155*^+/−^ ESC lines

Several categories of RNA species regulate a variety of cellular processes including pluripotency^13,14^. In particular, miRNAs exhibit tightly controlled dynamics to ensure pluripotent fidelity or differentiation commitment^15^. We focused our analysis on miRNAs that exhibited significantly altered transcript expression and found a total of 8 candidates enriched in our dataset (p < 0.05, fold change >2, Table 1). The rest of miRNAs included in our analysis did not show a significant change in expression between the *nup155*^+/−^ mouse ESCs and WT (Supplementary Fig. S4). The abnormally upregulated transcripts in this category consisted of *miR138–2*, a miRNA associated with nervous system development and regulation^16,17^, and 3 non-annotated BAC libraries transcripts. Downregulated miRNA transcripts included *miR291a*, *miR291b*, *miR293* and *miR294*, all of which belonged to the *miR290–295* cluster^18–20^.

**Table 1:** Significant changes in miRNAs of *nup155*^+/−^ ES cells compared to WT.

	Gene symbol	Gene description	Ensembl ID	FC	p Corr
Downregulated	*miR291a*	microRNA 291a	ENSMUSG00000078008	−3.27	0.00284
	*miR291b*	microRNA 291b	ENSMUSG00000078032	−2.65	0.00998
	*miR293*	microRNA 293	ENSMUSG00000078035	−2.06	0.01879
	*miR294*	microRNA 294	ENSMUSG00000077903	−2.22	0.03418
Upregulated	*AC153909.1*	—	ENSMUSG00000095596	2.08	0.00043
	*AC044807.1*	—	ENSMUSG00000092956	3.50	0.02382
	*AC138311.1*	—	ENSMUSG00000094381	3.42	0.04083
	*miR138-2*	microRNA 138-2	ENSMUSG00000065512	3.68	0.00392

Provided are the Gene Symbol, GENCODE Gene Description, Ensembl ID, Fold change (FC), and corrected p-value with false discovery rate (p Corr) for each miRNA that reached statistical relevance (p Corr < 0.05).

**Figure 3:**
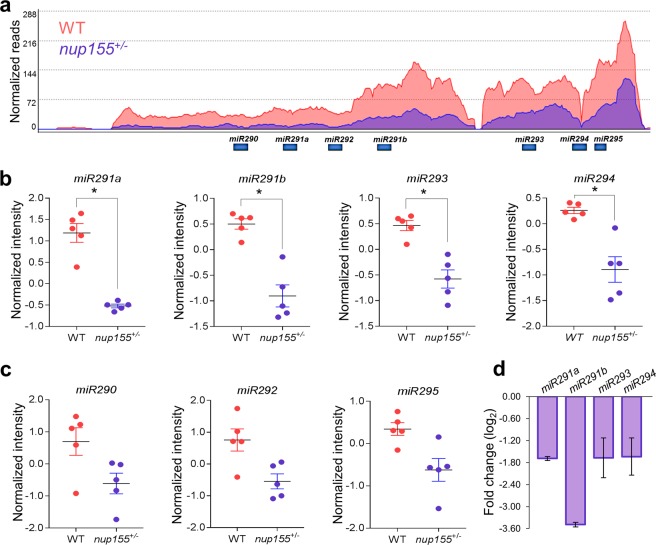
Nucleoporin insufficiency decreases ES cell expression of the *miR290–295* cluster. (**a**) RNAseq track data for WT (pink) and *nup155*^+/−^ (purple) ES cells showing diminished numbers of reads for the *miR290*–295 cluster. Members of the cluster include 7 miRNAs: *miR290, miR291a, miR291b, miR292, miR293, miR294*, and *miR295*. (**b**) Individual plots of the four statistically significant down regulated miRNAs identified in our dataset. (**c**) Remaining members of the *miR290–295* cluster recapitulate the down regulated trend, but did not reach statistical significance. Shown are changes for *miR290, miR292* and *miR295*. (**d**) Confirmation of down regulated expression by independent RT-qPCR. Bar graph shows decreased expression of *miR291a, miR291b, miR293*, and *miR294* in *nup155*^+/−^ cells compared to WT (*p < 0.05, n = 7).

Given the defined role of the *miR290–295* cluster in pluripotency^21–24^ we prioritized it for further investigation. Mapping normalized reads of the *miR290–295* cluster (Fig. 3a) shows a cluster wide decrease in transcription levels within *nup155*^+/−^ ESCs compared to wild type controls. Four of the seven member cluster were found to have statistically significant transcriptional downregulation (p < 0.05, fold change >2, Fig. 3b), while the remaining three miRNAs in the *miR209–295* cluster reflected the decreased transcription trend. (Fig. 3c). Validation of the RNAseq data by RT-qPCR confirmed diminished expression of *miR291a*, *miR291b*, *miR293* and *miR294* (p < 0.05, Fig. 3d).

### Expression of pluripotency factors is decreased in *nup155*^+/−^ embryonic stem cells

**Figure 4:**
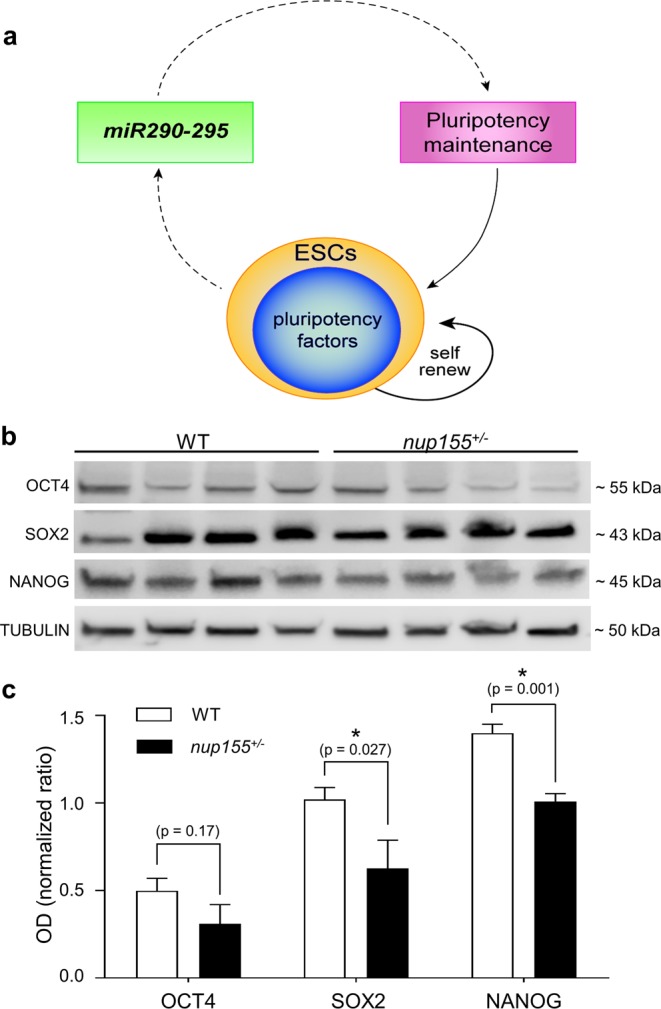
Decreased pluripotency factor protein expression in *nup155*^+/−^ cells. (**a**) Representation of the *miR290–295* cluster maintenance of pluripotency circuit in mouse ESCs, which is conducive to preservation of the self-renewal state of ESCs. (**b**) Western blots probing for OCT4, SOX2, and NANOG shows decreased protein expression in *nup155*^+/−^ cells relative to WT, β-tubulin was used as loading control. Shown are 4 biological replicates for WT and *nup155*^+/−^ ESC samples. Blots are shown as cropped images. Uncropped western blot images are included in Supplementary Files. (**c**) Densitometry readings for each lane were normalized to β-tubulin and analyzed using a two tailed, homoscedastic T-test. All three proteins in *nup155*^+/−^ cells exhibit decreased expression, with SOX2 (p = 0.027) and NANOG (p = 0.001) reaching statistical significance.

 The relationship between the *miR290–295* cluster and pluripotency^21–24^ (Fig. 4a) prompted us to examine the expression of *Oct4*, *Sox2* and *Nanog*. RT-qPCR results showed no changes in RNA expression of *Oct4* and *Sox2*, with significant downregulation of *Nanog* (n = 5, p < 0.05, Supplemental Fig. S5). At the protein level, immunoblotting revealed decreases in OCT4, SOX2 and NANOG expression in *nup155*^+/−^ cells (Fig. 4b). Densitometry analysis revealed downregulation in all three factors (Fig. 4c). OCT4 changes recapitulated the downregulated trend in the protein, while NANOG expression showed statistically significant change with 27.8% reduction in the protein from *nup155*^+/−^ ESCs compared to WT (n = 5 each condition, p < 0.01). SOX2 expression was also significantly decreased in *nup155*^+/−^ ESCs (38.5%, n = 5, p < 0.05, Fig. 4c).

**Figure 5:**
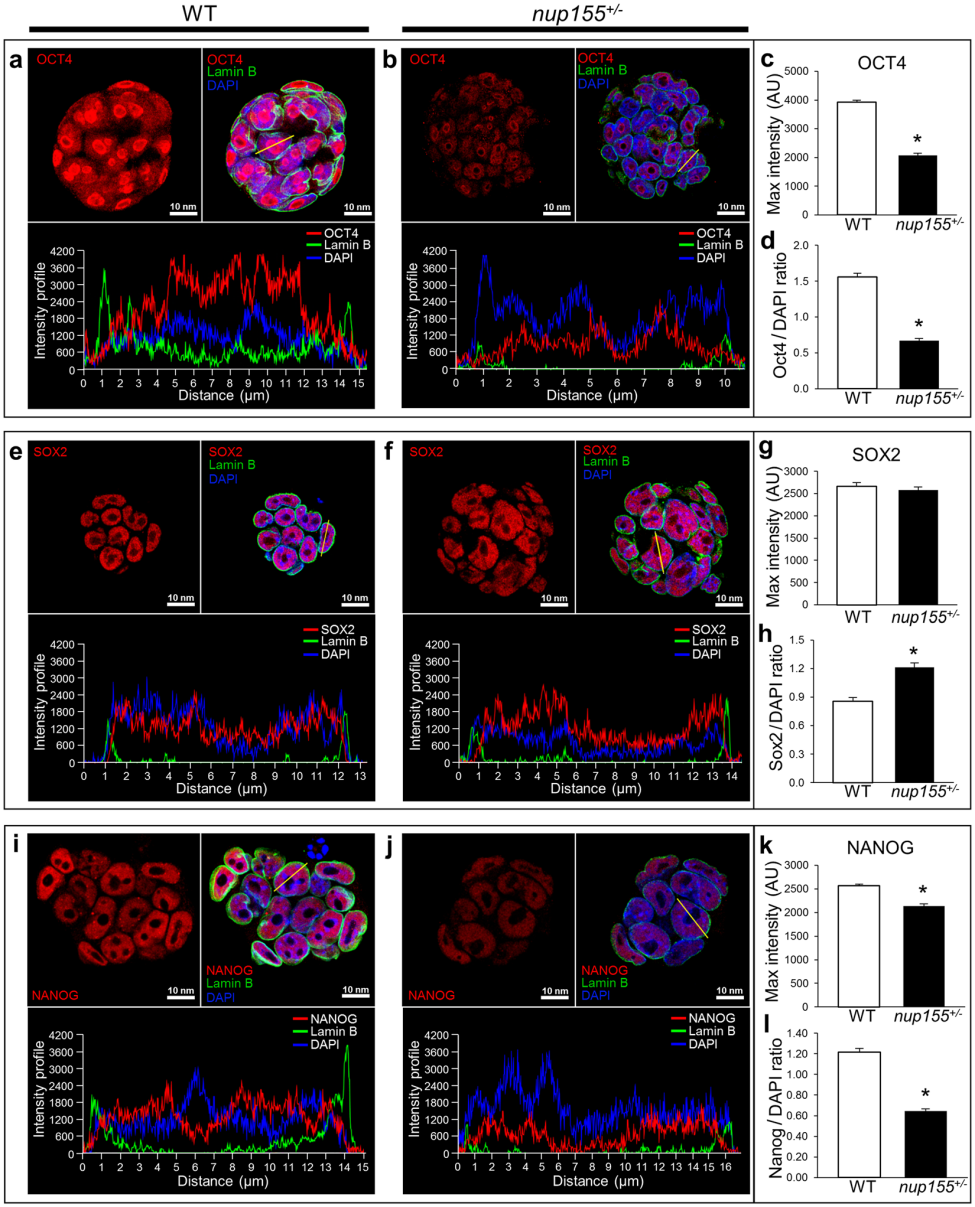
Expression of pluripotency factors is decreased in *nup155* deficient ESCs. (**a**,**b**) Representative images of OCT4 (red), Lamin B (green), and DAPI (blue) fluorescence signal depicting OCT4 intranuclear localization with overall decrease in signal profile in *nup155*^+/−^ (right panel) compared to ESC colonies. Bottom panel represents individual cell intensity profiles. (**c**,**d**) Maximum (Max) intensity of OCT4 in *nup155*^+/−^ ESC colonies was significantly decreased compared to WT, along with the OCT4:DAPI signal ratio (*p < 0.001). (**e,f**) SOX2 (red), Lamin B (green), and DAPI (blue) triple staining of *nup155*^+/−^ and WT ESC colonies show no overall change in intranuclear signal profile, as well as (**g**) no change in SOX2 max signal intensities between conditions (p = 0.42). (**h**) SOX2:DAPI signal ratio is higher in NUP155 deficient cells compared to WT. (**i,j**) NANOG (red) fluorescent signal profile was diminished in *nup155*^+/−^ compared to WT. (**k**,**l**) NANOG (red) max intensity and NANOG:DAPI ratio were significantly decreased in *nup155*^+/−^ ESC colonies compared to WT (*p < 0.001).

Immunocytochemistry analysis revealed that protein localization of these core pluripotency factors within ESCs was stable in *nup155* deficient and WT conditions, depicting robust nuclear localization for SOX2, OCT4 and NANOG with clear exclusion from nucleoplasmic DAPI negative regions (Fig. 5a,b,e,f,i,j). Significantly, analysis of the signal intensity profile^25–28^ revealed that maximum signal intensity was significantly diminished for OCT4 and NANOG in *nup155* deficient cells compared with WT (n = 53 and n = 51 respectively; p < 0.001; Fig. 5c,k), with no significant differences observed for Sox2 (Fig. 5g). Ratiometric comparison of the maximum signal between each factor to DAPI fluorescent signal was significantly changed in *nup155*^+/−^ cells compared to WT (p < 0.05; Fig. 5d,h,l). Overall these data are in line with the immunoblot data showing diminished protein expression for the three pluripontency factors.

## Discussion

Nucleoporins regulate development and disease by a diversity of putative epigenomic mechanisms that may be employed to regulate stem cell pluripotency. In the present study, dysregulated expression of a variety of ncRNA species occur in a nucleoporin insufficient stem cell genomic background. Specifically, discrete classes of ncRNA are abnormally expressed in ESCs which harbor a *nup155* disruption with the *miR290–295* cluster significantly downregulated in a heterozygous *nup155*^+/−^ line. *In vitro*, these cells exhibit diminished proliferation accompanied by decreased expression of pluripotency factors that are part of a regulatory circuit that includes the *miR290–295* cluster. This study is the first to demonstrate downstream effects for *nup155* on *miR290–295* cluster expression and implicates a potential pathway by which nups regulate pluripotency through effects on ncRNA expression.

Building upon our previous work that identified transcriptome remodeling of the pro-arrhythmogenic *nup155*^+/−^ ESC line^7,8^, our findings here reveal a pluripotency-regulating non-coding RNA cluster downstream of *nup155* gene disruption. The impaired ESC colony characteristics observed are supported by enrichment in specific downregulation of the *miR290–295* cluster in our analysis. This is significant given that the *miR290–295* family is the most abundant miR cluster in ESCs, and its members underlie self-renewing functions of pluripotent cells^18,21^. Deficient nucleocytoplasmic transport is a key feature of *nup155* disruption^7^, thus specific targeting of *miR290–295* cluster expression in a *nup155* deficient ESC line may be due to diminished nuclear localization of OCT4 (Fig. 5a–d). This is in addition to overall decreases in pluripotent factor expression (Fig. 4). Diminished nuclear OCT4 may thus uncouple the pluripotent regulatory circuit consisting of the *mirR290-295* cluster and OCT4^18^, potentially exacerbating *miR290–295* cluster down regulation in *nup155*^+/−^ ESCs.

In the present study, downregulation of the *miR290–295* cluster was associated with a decrease in OCT4, SOX2 and NANOG, canonical markers of pluripotency^15,18,21,23^. This manifested as an overt reduction in cell proliferation and ESC colony size despite limited reduction in OCT4 and SOX2, in line with the notion that other regulatory mechanisms may contribute to overall ESC phenotype^18,29^. For example, recent work reported a critical role for *miR294* expression and posttranscriptional dynamics in remodeling the gene regulatory network of ESCs^30^. In their study, the authors demonstrated that crosstalk between *miR294* and the splicing factor Mbnl1/2 controlled global alternative splicing in ESCs. Furthermore, *miR294* acted directly, through targeting Mbnl1/2 RNA by *miR294*, as well as epigenetically by facilitating recruitment of the repressor PRC2 to the promoter region of *mbnl1/2*^30^.

The effects of a *nup155* gene lesion on pluripotency is supported by previous work in other nups that have demonstrated functional roles in stem cell regulation and fate selection^31–33^. For example, regulation of embryonic stem cell pluripotency has been demonstrated for NUP153 and its ability to discretely silence chromatin^33^. This was further elaborated on in a model of neural development, where it was discovered that NUP153 interacted with the pluripotency factor SOX2 to maintain neural progenitors in an undifferentiated state^31^. Similarly, work in models of myogenesis demonstrated a role for NUP210/GP210 in myotube differentiation^34^, and how NUP210 mediated differential expression of several genes that controlled muscle cell maturation.

Given the unique property of stem cells to self-renew and differentiate into cells of different lineages, their capacity for fate specification under conditions of nup deficiency may be impacted. With respect to this, we investigated *lin28* and *let-7*, elements of an evolutionarily conserved heterochronic signaling pathway that governs differentiation in embryonic stem cells^35–39^. In our study, we detected transcriptional expression of *lin28a, lin28b, let7d* that was stable and did not significantly change in a nup155-deficient background (Supplementary Fig. S6). Conversely, *let7c-2* was downregulated by 1.17 fold (p = 0.02) (Supplementary Fig. S6). Together, these results suggest that the overall ability of *nup155*^+/−^ ESCs to acquire and secure distinct cell fates is preserved, albeit with some level of impairment. This is supported by our previous study in which *nup155*^+/−^ ESCs were able to spontaneously form embryoid body-derived cardiomyocytes, though with dysregulated electrical function^8^. These findings are in line with independent work that has reported similar results^7^. This retained ability to differentiate may thus reflect pluripotent cell sensitivity to proliferative, rather than specification, impairments caused by nup deficiency^5,34,40,41^.

From our previous study, we found transcriptome changes occured independent of differential subcellular compartmentalization, as total RNA extracted from ESCs represents absolute RNA expression independent of subcellular origin^8^. These expression differences may reflect an uncharacterized epigenomic function of NUP155 to regulate transcription levels, as similar functions for other nups have been independently characterized^31,33,34^. It is important to note that these changes may occur concomitant with dysregulated mRNA transport, as differential mRNA transport under conditions of *nup155* insufficiency may contribute to altered protein expression^7,10,42^. The precedence for chromatin-directed pre-transcriptional control mediated by NUP155 is supported by previous work in yeast and fly models of gene regulation. For example, the NUP155 homolog NUP170 has been shown to bind telomeric and subtelomeric regions to regulate both nucleosome positioning and chromatin silencing^43^. Another NUP155 homolog, NUP157, binds to chromatin independent of nucleotide sequence^44^. Specifically, the crystal structure of recombinant NUP157 was found to form a C-shaped conformation with a large patch of positively charged residues asymmetrically distributed on its surface that mediated chromatin interactions^44^. Consistent with this, recombinant NUP157 bound both DNA and RNA in a sequence independent manner comparable to other proteins with similarly charged structures^44^. In *Drosophila* nurse cells, NUP155 functions as part of a negative regulatory loop that acts in concert with other NUPs of the nuclear pore complex to control chromatin organization and gene activation/repression^45^. In this model, discrete regions of chromatin are tethered to the nuclear periphery via interactions with NUP155. Chromatin disengages from the nuclear envelope and re-localizes to the nucleoplasm when NUP155 interacts with other NUPs within the pore, thus alleviating gene silencing at the nuclear periphery and effecting gene activation^45^.

Alternatively, indirect chromatin interactions of NUP155 may explain its gene regulatory effects. In a model of cardiac hypertrophy, NUP155 was immunoprecipitated with repressive histone deacetylases (HDACs), in line with the gene silencing environment at the nuclear periphery^9,46,47^. In this study by Kehat *et al*., protein domain mapping and biochemical characterization confirmed physical interactions of NUP155 and HDAC4. Furthermore, measuring gene expression changes in downstream HDAC4 targets provided functional validation of the NUP-HDAC interaction^9^. More work is necessary to define the role of NUP155 in distinct cellular contexts as well as reconcile its implicated dual gene activation and repression functions.

The identification here of a nup-miRNA signaling axis suggests that nup regulation of gene expression may not only be restricted to promoter-directed mechanisms. In the absence of conspicuous canonical markers of cardiac disease, regulated miRNA expression during cell fate specification may be a contributing mechanism involved in nup-associated cardiopathology. Thus, while the role for miRNA regulation in cardiac development is well understood^48–51^, the functional relationship of nups and miRNAs may be a contributing and underlying mechanism to explain the idiopathic atrial fibrillation phenotype associated with *nup155* deficiency.

## Methods

### Embryonic stem cell culture

Wild type (WT) and NUP155 exon truncated E14TG2a.4 (*nup155*^*+/−*^) feeder independent mouse ESC lines were cultured on 0.1% gelatin coated 100 mm dishes grown in 10 ml of 2i media consisting of 95% Glasgow MEM (GMEM), 5% ES qualified fetal bovine serum, sodium pyruvate, non-essential amino acids, penicillin/streptomycin, β-mercaptoethanol, ESGRO leukemia inhibitory factor (LIF), GSK-3 Inhibitor XVI and MEK1/2 Inhibitor III. After initial plating (seeding density between 3.0 × 10^6^ and 5.5 × 10^6^ cells), cells were maintained in culture for 2–3 passages, changing 2i media as required. At approximately 80% confluency, cells were passaged by treatment with 5 ml of 0.25% trypsin for 4 min at 37 °C. Trypsin digestion was arrested by addition of equal parts 2i media. This suspension was centrifuged at 1500 rpm for 4 min and resuspended in 1 mL of 2i media for cell counting (Countess II Automated Cell Counter, Life Technologies Corporation, Carlesbad, CA) and expansion.

### ESC colony characterization

ESC culture was stained for alkaline phosphatase^52^ according to manufacturer protocol (Millipore, Burlington, MA). Briefly, fixed cells were incubated for 15 min. with staining solution that included fast red violet, naphthol AS-BI phosphate solution and water at 2:1:1 ratio. Following staining the cells were imaged on an Olympus IX71 microscope using a 4x objective. Images were stored as high resolution TIFF files and analyzed using the IMJ Edge macro in ImageJ/Fiji^53^. Absoute pixel-based measurements of diameter and percent area were made for WT and *nup155*^+/−^ ESC colonies. Statistical significance was determined using Student’s T-Test, with p-value ≤ 0.05 set as threshold. A total of 3 independent replicates were imaged for each ESC line.

### RNA extraction and PCR validation

Cells were passaged as described above and suspended in PBS prior to RNA extraction. Approximately 1.0 × 10^6^ cells from murine embryonic stem cells were harvested and total RNA was isolated as previously described^8^. To confirm expression of miRNAs in the *miR290–295* cluster as well as upstream pluripotency factors Sox2, Oct4 and Nanog, reverse transcriptase quantitative polymerase chain reaction (RT-qPCR) was performed. Total RNA was converted into cDNA using TaqMan MicroRNA (Applied Biosystems, Foster City, CA) or SuperScript IV VILO (Applied Biosystems, Foster City, CA) kits for miRNA or gene expression assays, respectively. Quantitative real-time polymerase chain reaction (qPCR) was performed using TaqMan Fast Advanced Master Mix (Applied Biosystems, Foster City, CA). Gene expression levels were quantified using the 7500 Real-Time PCR System (Applied Biosystems, Foster City, CA). Expression levels were determined using the 2^−ΔΔCt^ method^54^, with *snoRNA429* (Applied Biosystems, Foster City, CA, Cat. #: 4427975) and *Gapdh* (Applied Biosystems, Foster City, CA, Cat. #: 4351370) used as respective housekeeping genes for miRNA and pluripotency factors *Sox2, Oct4*, and *Nanog*.

### RNA-seq and Bioinformatic analysis

RNA-seq analysis using Strand NGS (Agilent Technologies, Santa Clara, CA) was performed previously (NIH GEO database accession number GSE111596)^8^. For the present analysis, transcriptome data from GSE111596 was realigned with Ensembl prior to analysis for non-coding transcripts. Using the ‘Translate Genes to Regions’ utility in Strand NGS, a total of 8822 unique genic regions were identified. These were divided into 4278 upregulated and 4544 downregulated unique regions that were classified into exonic, intronic, intergenic regions. Further subcategorization into different ncRNA subtypes included long non-coding RNAs, antisense RNAs, miRNAs, small nucleolar RNAs, small nuclear RNAs, ribosomal RNAs, sense overlapping transcripts, miscellaneous or unknown ncRNAs. These ncRNAs were cross referenced with the mouse ncRNA identities from GENCODE annotation database (version 17, https://www.gencodegenes.org/).

### Western blot

Protein expression of NUP155, SOX2, OCT4 and NANOG was determined by western blot. Total protein was isolated from WT and *nup155*^+/−^ murine ESCs using RIPA buffer. Four biological replicates of each cell type were loaded onto a 4–20% SDS PAGE gradient gel with 32 ng protein loaded per well. Following electrophoresis (100 V for ~1 hr), proteins were transferred to PVDF membrane using the iBlot2 Dry Blotting System (BioRad Laboratories, Hercules, CA). Membranes were blocked at room temperature in 5% milk in TBST for 1 hour before overnight incubation at 4 °C with primary antibodies diluted in 1% milk in TBST. NUP155, SOX2, OCT4, NANOG and β-tubulin antibodies were used at concentrations specified in Supplementary Table S2. Secondary antibody incubation was performed with HRP-conjugated antibodies (Supplementary Table S2). Membranes were developed with SuperSignal® West Pico Chemiluminescent Substrate (Thermo Fisher Scientific, Waltham, MA) for 5 minutes and imaged using the Odyssey Fc imaging system with Image Studio v5.2 (LI-COR Biosciences, Lincoln, NE). Densitometric analysis was performed using ImageJ and analyzed with GraphPad Prism 7 software (San Diego, CA). SOX2, OCT4 and NANOG were normalized to β-tubulin loading controls. The four biological replicates from *nup155*^+/−^ and WT ESCs were compared using a two tailed, homoscedastic T-test, and significance was set at p-value < 0.05.

### Immunocytochemistry and Image analysis

Cells cultured in chambered coverslips (Thermo Scientific^®^ Nunc^®^ Lab-Tek II, Waltham, MA) were pre-fixed for 2 min with 4% paraformaldehyde in culture media, then fixed with 2% PFS for 20 min at room temperature and stored with 1x phosphate buffered saline (PBS) at 4 °C until use. Before staining, all cells were permeabilized (0.3% Triton^®^ X-100 in PBS) for 15 min, then blocked for 45 min in blocking serum consisting of 5% normal donkey serum and 1% bovine serum albumin (BSA) in PBS plus Tween-20 (PBS-T). Staining with primary antibodies (diluted in 1% BSA in PBS-T) was performed by overnight incubation at 4 °C in humidified dark chambers. SOX2, OCT4, NANOG and Lamin B antibodies were used at concentrations specified in Supplemental Materials (Supplementary Table S2). Following staining with primary antibodies, cells were washed with PBS-T and incubated for 1 hour at room temperature with secondary antibodies (Supplementary Table S2). After a 6 min wash with PBS-T, cells were counterstained with 4′,6-diamidino-2-phenylindole (DAPI, 1 µg/ml) for 10 minutes, then rinsed once with PBS, once with deionized water and air dried for 2 min. ProLong Diamond mounting media (Thermo Fisher Scientific, Waltham, MA) was added to each chambered coverslip and cured for 24 hrs at room temperature before imaging. Stained samples were imaged with Nikon A1R confocal (Nikon Corporation, Tokyo, Japan), using a 100x oil-objective and 2x digital zoom. Each comparing set of samples stained with each antibody was imaged the same day.

Image analysis of ESC colonies was done using NIS-Elements AR imaging software v4.20 (Nikon Corporation, Tokyo, Japan). Signal intensity profile from each individual ESC was extracted, taking care that measurements were along the widest part of the cell, including the entire nucleus and at least one nucleolus. Intranuclear maximum (Max) signal intensity was defined for each fluorescent signal, SOX2, OCT4 or NANOG (594 nm, red) and DAPI (405 nm, blue), based on Lamin B (488 nm, green) signal.

## Supplementary information

Supplementary Material

## Data Availability

All data generated or analysed during this study are included in this published article (and its Supplementary Information Files).
