# Supplementary material for: Nucleoporin insufficiency disrupts a pluripotent regulatory circuit in a pro-arrhythmogenic stem cell line
Source: Sci Rep. 2019 Sep 3;9:12691. doi: 10.1038/s41598-019-49147-4 (PMC6722237; doi:10.1038/s41598-019-49147-4)
Supplement: Supplementary file 1 — Supplementary Material [file 41598_2019_49147_MOESM1_ESM.pdf]

Nucleoporin insufficiency disrupts a pluripotent regulatory circuit in a pro-arrhythmogenic stem cell line

Claudia C. Preston[1‡], Emily C. Storm[1,‡], Ryan D. Burdine[1], Tyler A. Bradley[1], Andrew D. Uttecht[1], Randolph S. Faustino[1,2*]

[1]Genetics and Genomics Group, Sanford Research, 2301 E. 60th Street N., Sioux Falls, SD 57104, [2]Department of Pediatrics, Sanford School of Medicine of the University of South Dakota, 1400 W. 22nd Street, Sioux Falls, SD 57105

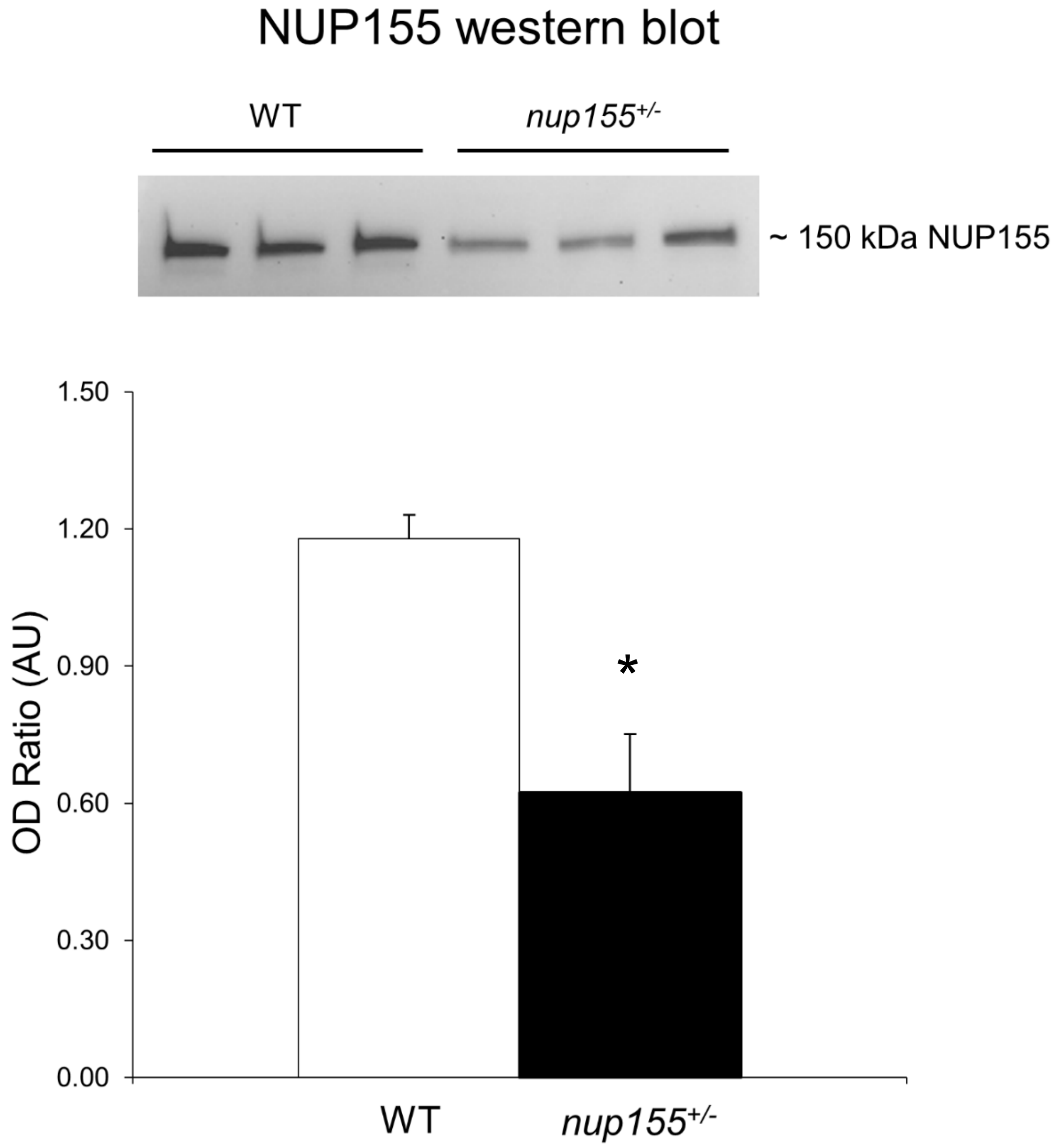

Supplementary Figure S1: Protein expression of NUP155 in mouse ESCs. Depicted is a western blot image (top) of NUP155 (band seen at ~150kDa) in WT (n=3) and *nup155*$^{+/-}$ (n=3) ESCs. Densitometric analysis (performed using Image J and normalized to β-tubulin) shows significant decrease in expression in *nup155*$^{+/-}$ ESCs (47%) when compared to WT. * = $p < 0.05$. Uncropped western blot image is included in Supplementary Fig. S7.

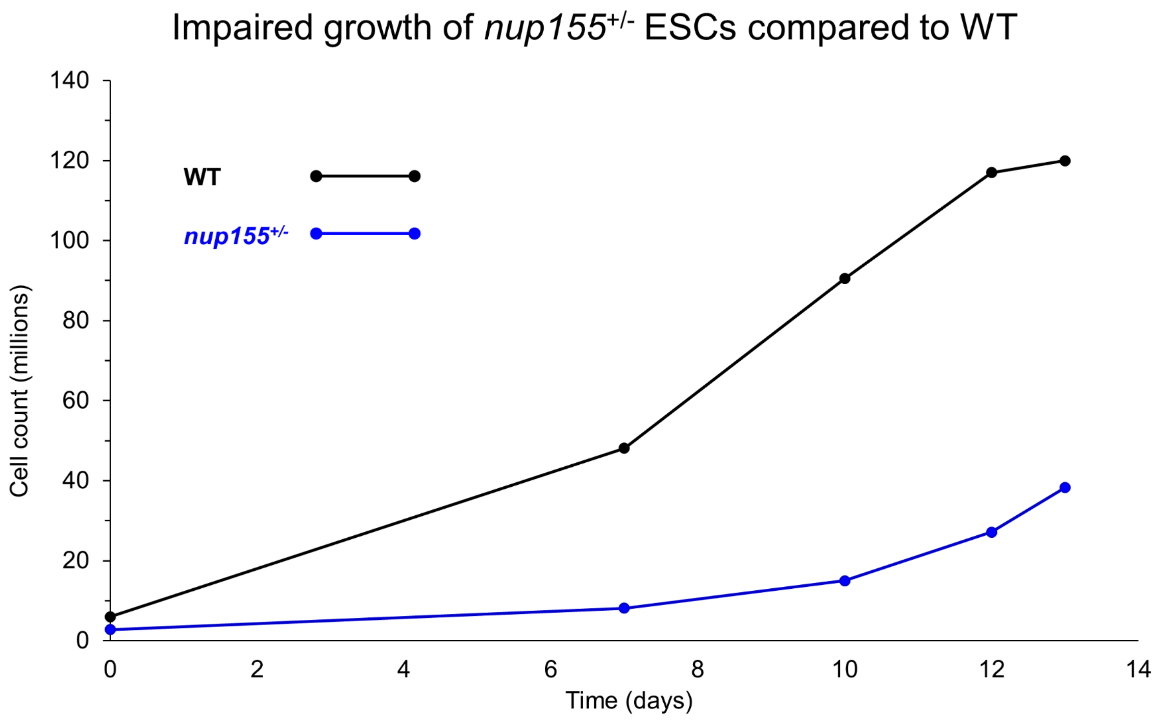

Supplementary Figure S2: Growth curve of mouse embryonic stem cells. (ESCs) shows impaired growth in *nup155*$^{+/-}$ cells compared to WT controls. Y-axis depicts the live cell count in millions, determined by Trypan Blue exclusion assays for cell viability. Cell counts were performed at time of passaging during four consecutive passages of WT ES cells (black) and *nup155*$^{+/-}$ ES cells (blue).

Categorization of upregulated non-coding RNAs in *nup155*$^{+/-}$ ESCs

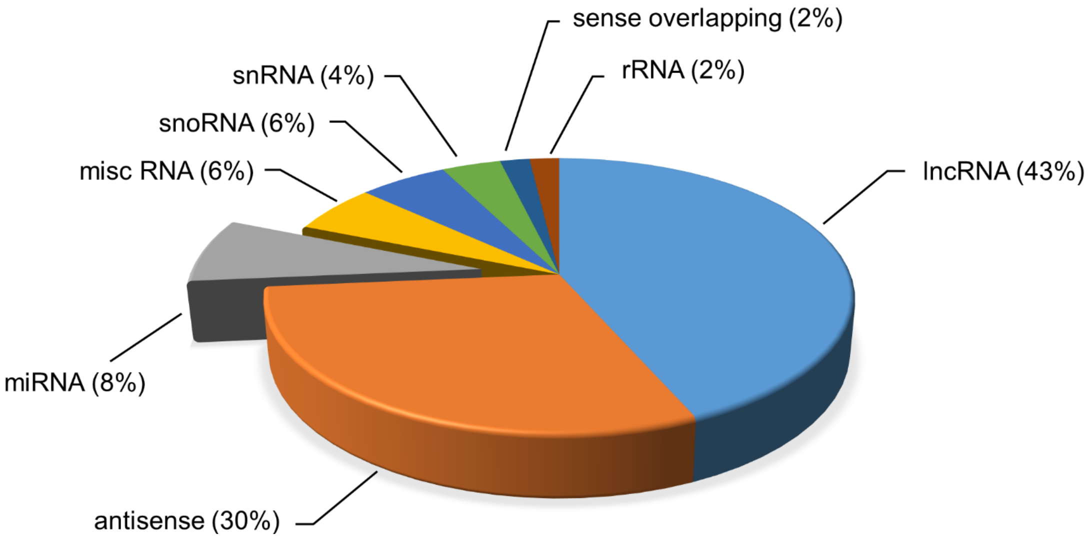

Supplementary Figure S3: Categorization of upregulated non-coding RNAs**.** Pie chart detailing percent composition of statistically significant upregulated non-coding RNAs (defined as corrected p value < 0.05) from RNAseq. There were a total of 53 significantly upregulated non-coding RNAs including 23 long non-coding RNAs (lncRNA), 16 anti-sense RNAs (antisense), 4 microRNAs (miRNA), 3 miscellaneous non-coding RNAs (misc RNA), 3 small nucleolar RNAs (snoRNAs), 2 small nuclear RNAs (snRNAs), 1 ribosomal RNA (rRNA), and 1 sense overlapping transcript. Entities categorized as pseudogenes and processed transcripts were omitted for clarity.

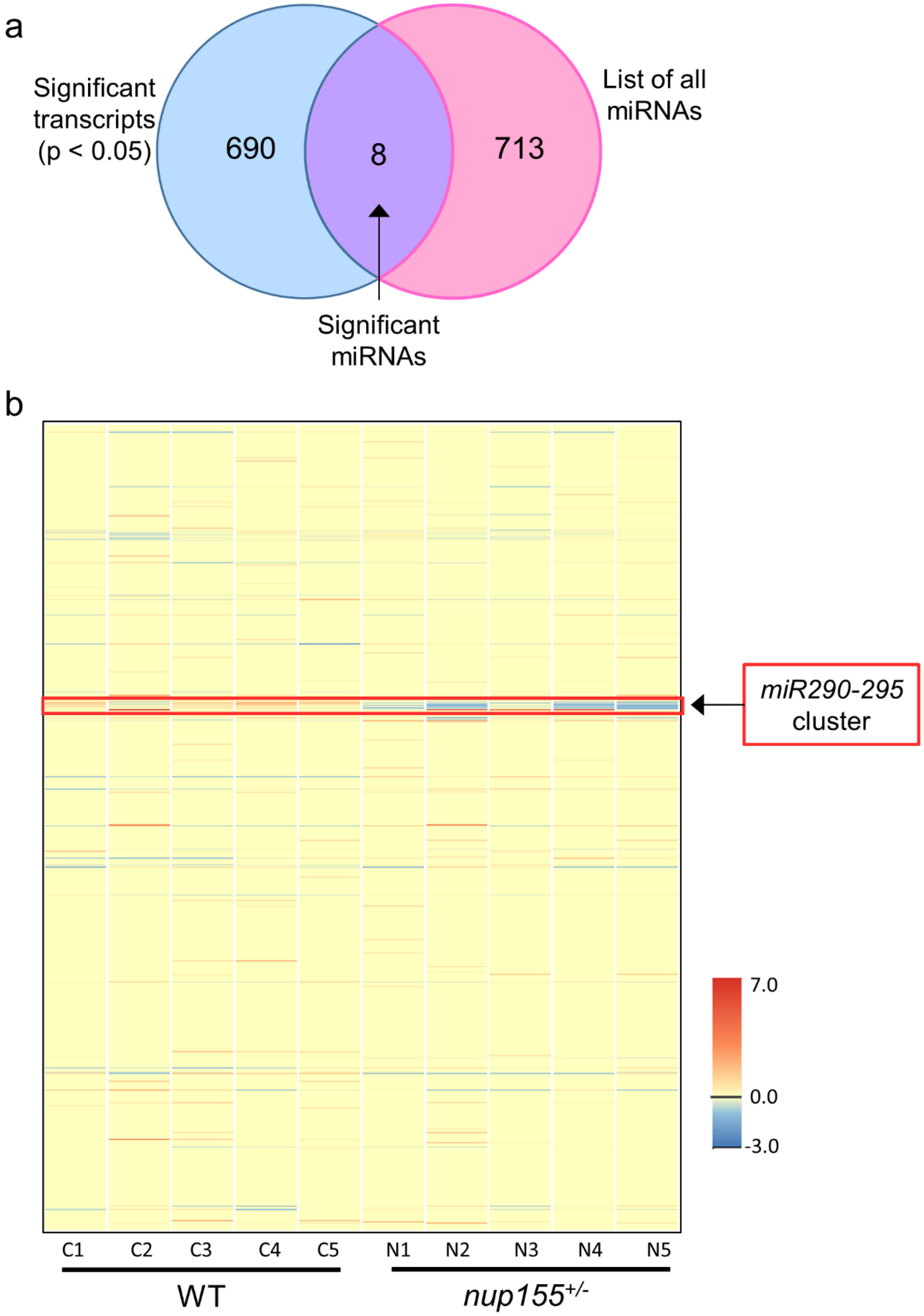

Supplementary Figure S4: Transcriptional signature of miRNAs in *nup155*$^{+/-}$ mouse ESCs. (a) Venn diagram depicting total significant transcripts in our dataset (698) intersected with all miRNAs present (721). Total significant miRNAs changing in *nup155*$^{+/-}$ ESCs compared with WT as indicated. (b) Heatmap representing expression of all 721 miRNAs between WT (n = 5) and *nup155*$^{+/-}$ (n = 5) ESC samples. Highlighted (red box) are the members of the *miR290-295* cluster. Color legend shows normalized fold change expression from -3.0 (blue) to 7.0 (red).

Decreased transcription of pluripotency factors in *nup155*$^{+/-}$ cells

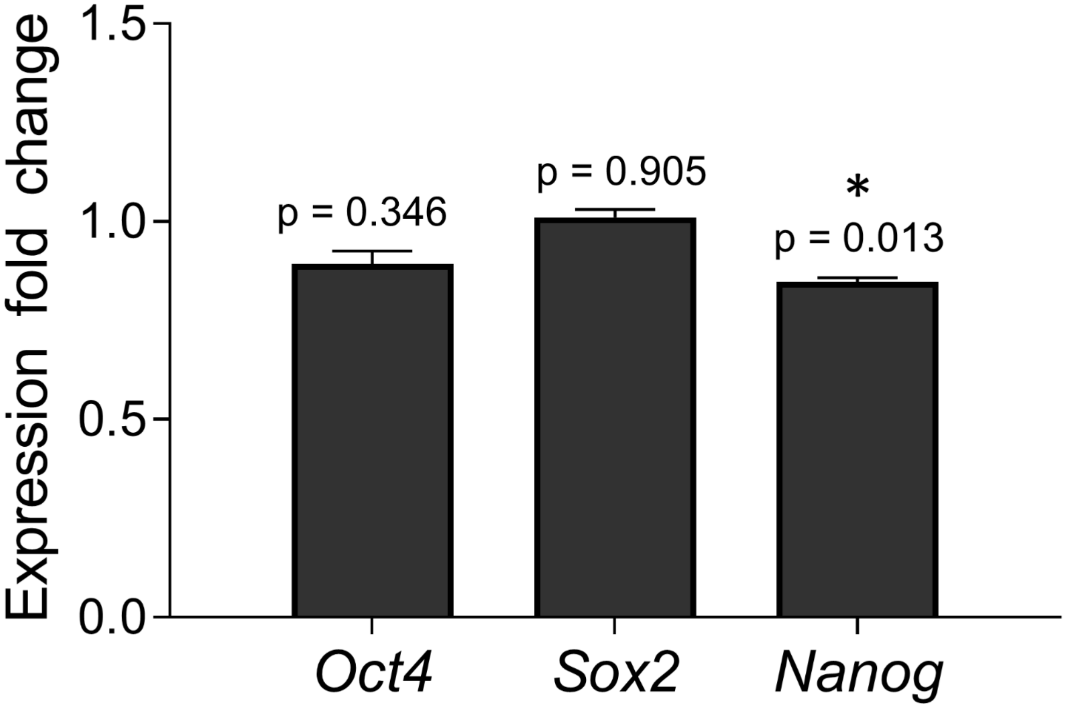

Supplementary Figure S5: Expression fold change of pluripotency factors in *nup155*$^{+/-}$ cells relative to WT. Graph depicts expression fold change ($2^{-\Delta\Delta Ct}$) of pluripotency factors *Sox2*, *Oct4* and *Nanog* transcripts measured by RT-qPCR (WT n = 5; *nup155*$^{+/-}$ n = 5). Bar graph shows decreased expression of *Nanog* ($p = 0.0131$) in *nup155*$^{+/-}$ ES cells compared to their wild type counterparts. *Sox2* and *Oct4* transcripts did not change to a statistically significant degree. Significance was determined as $p < 0.05$.

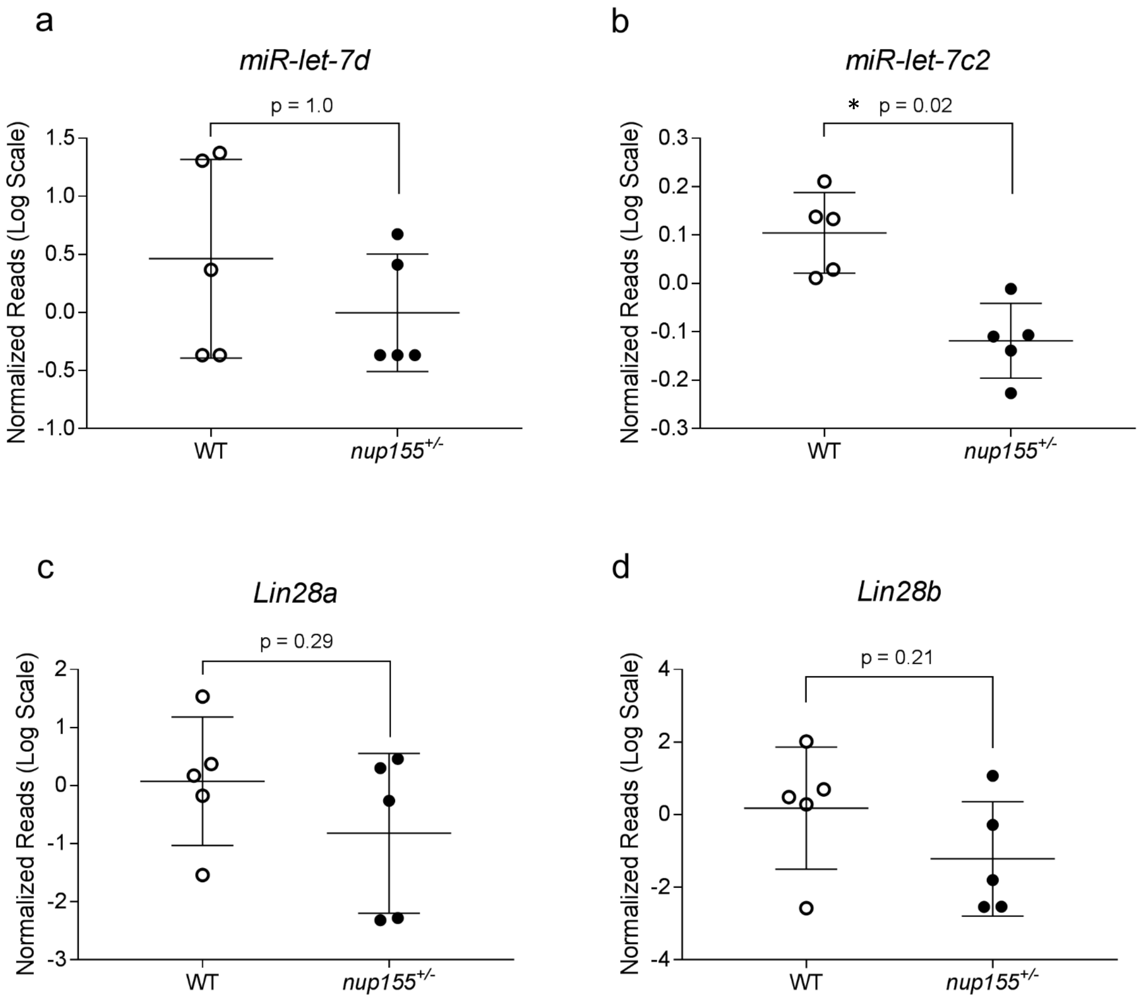

Supplementary Figure S6: Expression of *let7* family members. Non-significant normalized reads of *miR-let-7d*, *Lin28a* and *Lin28b* in WT and *nup155*$^{+/-}$ ESCs ($p > 0.05$). Expression of *miR-let-7c2* in *nup155*$^{+/-}$ ESCs was significantly decreased when compared to WT (fold change 1.17, $p = 0.02$).

Uncropped western blot images

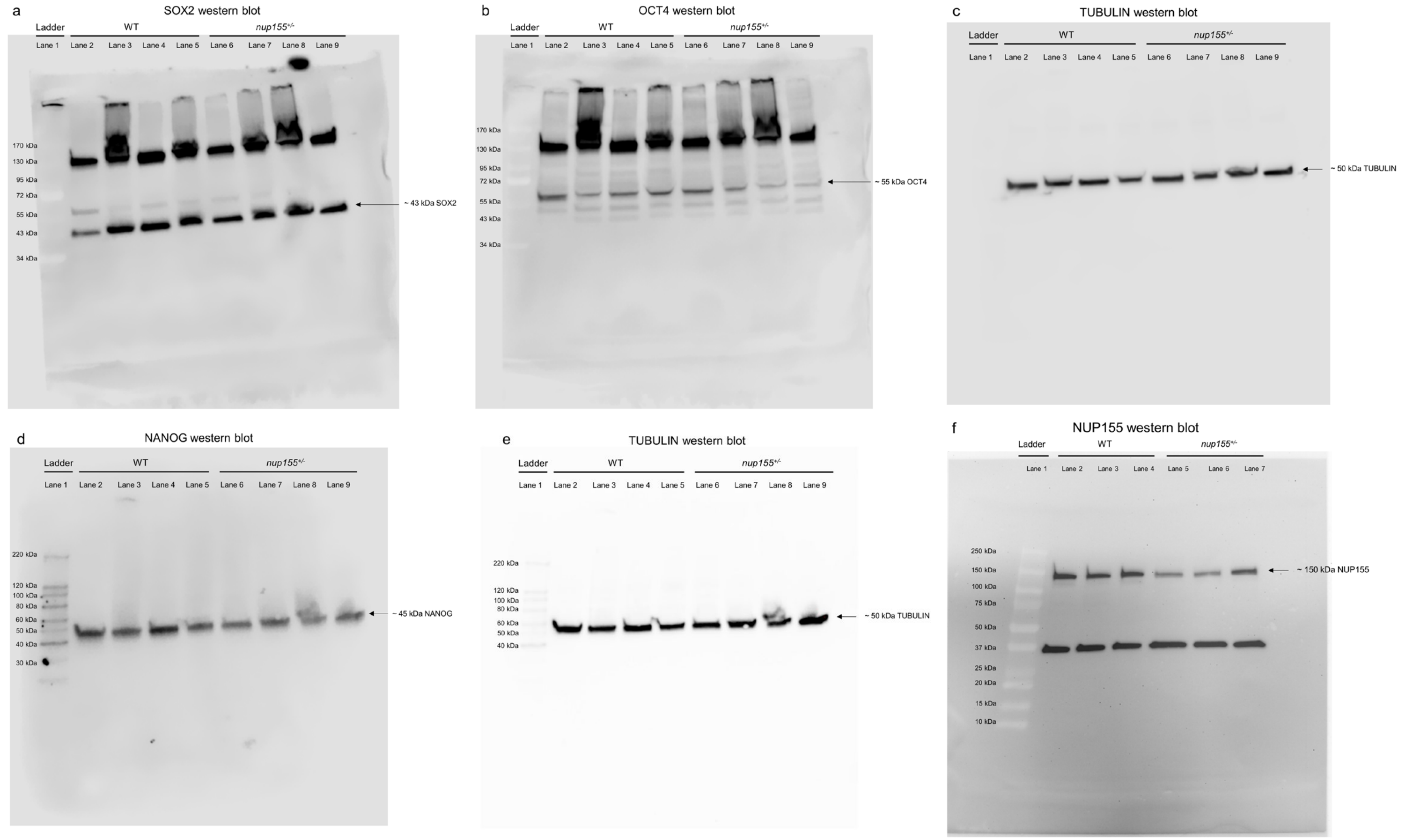

Supplementary Figure S7: Western blot images of mouse ESCs. WT (lanes 2-5) and *nup155*$^{+/-}$ (lanes 6-9) were run on a 4-10% gradient gel (100V, 60 min) with EZ-run protein ladder (lane 1) and probed for (a) SOX2 (band seen at ~43 kDa) then stripped and re-probed for (b) OCT4 (band seen at ~55 kDa). Blot was imaged individually with HRP conjugated secondary antibody. The membrane was stripped a third and final time and probed for (c) β-tubulin (50 kDa) for use as a loading control. A duplicate gel was run with MagicMark XP ladder (lane 1), and immunoblotted for (d) NANOG (~45 kDa), then stripped and probed for (e) β-tubulin (50 kDa). (f) Uncropped western blot image of NUP155 is seen here depicting WT samples in lanes 2-4 and *nup155*$^{+/-}$ in lanes 5-7 (Precision Plus Dual Color protein ladder in lane 1), with a predicted band at ~150 kDa. The blot was then stripped and probed for β-tubulin as loading control. Densitometric analysis was performed using ImageJ. OCT4, SOX2, NANOG and NUP155 were normalized to β-tubulin on their respective blots.

Supplementary Table S1: Significantly altered non coding RNAs in *nup155*$^{+/-}$ ESCs

	Gene Type	Gene Symbol	Description	Ensembl ID	FC	p Corr
Upregulated	lincRNA	*D430040D24Rik*	RIKEN cDNA D430040D24 gene	ENSMUSG00000087589	1.4724681	0.001178881
	lincRNA	*D330023K18Rik*	RIKEN cDNA D330023K18 gene	ENSMUSG00000087269	1.7457452	0.005554195
	lincRNA	*Gm14321*	predicted gene 14321	ENSMUSG00000084797	1.4820379	0.01455762
	lincRNA	*RP23-404J7.4*	-	ENSMUSG00000097539	3.1235678	0.011057199
	lincRNA	*RP23-404J7.5*	-	ENSMUSG00000097299	3.0020168	0.024969164
	lincRNA	*AC136729.1*	-	ENSMUSG00000097183	1.3317534	0.001000993
	lincRNA	*Gm11827*	predicted gene 11827	ENSMUSG00000086765	1.1660783	0.042046305
	lincRNA	*Gm12503*	predicted gene 12503	ENSMUSG00000087326	1.7530234	0.004078499
	lincRNA	*Abhd1*	abhydrolase domain containing 1	ENSMUSG00000006638	1.6989294	0.01895062
	lincRNA	*Wbscr25*	Williams Beuren syndrome chromosome region 25 (human)	ENSMUSG00000054909	1.7417476	0.032269794
	lincRNA	*AC164564.1*	-	ENSMUSG00000097079	1.579294	0.00063897
	lincRNA	*AC140392.2*	-	ENSMUSG00000096976	1.9289386	0.003545671
	lincRNA	*AC124662.1*	-	ENSMUSG00000097223	1.2479576	0.008354949
	lincRNA	*AC152058.2*	-	ENSMUSG00000097013	1.1873827	0.006195859
	lincRNA	*AC152410.1*	-	ENSMUSG00000097889	2.7549548	0.023123108
	lincRNA	*Gm11431*	predicted gene 11431	ENSMUSG00000087407	3.2660005	0.00802013
	lincRNA	*Gm11769*	predicted gene 11769	ENSMUSG00000085636	1.2769991	0.000249433
	lincRNA	*AC138303.1*	-	ENSMUSG00000097902	3.051519	0.00016714
	lincRNA	*AC134826.1*	-	ENSMUSG00000097709	2.6784124	0.023095299
	lincRNA	*AC116484.1*	-	ENSMUSG00000097867	1.1609259	0.015857091
	lincRNA	*AC122247.1*	-	ENSMUSG00000097318	1.1536374	0.001217811
	lincRNA	*AC163347.1*	-	ENSMUSG00000096948	2.851541	0.001072407
	lincRNA	*1600025M17Rik*	RIKEN cDNA 1600025M17 gene	ENSMUSG00000085114	2.171279	0.02288163
	antisense	*Gm13748*	predicted gene 13748	ENSMUSG00000086836	1.7096071	0.027327938
	antisense	*Gm13415*	predicted gene 13415	ENSMUSG00000085057	1.3930378	0.003980869
	antisense	*Gm13704*	predicted gene 13704	ENSMUSG00000086544	1.0402733	0.049498916
	antisense	*1700024P03Rik*	RIKEN cDNA 1700024P03 gene	ENSMUSG00000086186	1.5176938	0.029582342
	antisense	*5430417L22Rik*	RIKEN cDNA 5430417L22 gene	ENSMUSG00000074918	1.3054546	0.008127411
	antisense	*Gm14097*	predicted gene 14097	ENSMUSG00000084839	1.4925072	0.000779107
	antisense	*Gm16316*	predicted gene 16316	ENSMUSG00000087129	1.2248495	0.017290777
	antisense	*Gm17112*	predicted gene 17112	ENSMUSG00000090873	1.6337795	0.008538174
	antisense	*Fcor*	Foxo1 corepressor	ENSMUSG00000089665	1.0110854	0.020920018
	antisense	*Gm15816*	predicted gene 15816	ENSMUSG00000086741	1.6828485	0.03832272

	antisense	*Gm12228*	predicted gene 12228	ENSMUSG00000086789	1.1755149	0.000258036
	antisense	*Gm12298*	predicted gene 12298	ENSMUSG00000072834	1.4753325	0.031967454
	antisense	*Gm15911*	predicted gene 15911	ENSMUSG00000086506	1.7776895	0.029089505
	antisense	*Gm4524*	predicted gene 4524	ENSMUSG00000090257	1.8096772	0.003922481
	antisense	*1700062I23Rik*	RIKEN cDNA 1700062I23 gene	ENSMUSG00000086898	2.6986656	0.009683897
	antisense	*Gm16538*	predicted gene 16538	ENSMUSG00000089766	1.3301628	0.004375643
	miRNA	*AC153909.1*	predicted gene, GM24303	ENSMUSG00000095596	1.058854	0.000426411
	miRNA	*AC044807.1*	predicted gene, Gm26220	ENSMUSG00000092956	1.805843	0.023821399
	miRNA	*AC138311.1*	predicted gene, Gm25068	ENSMUSG00000094381	1.7731758	0.040833537
	miRNA	*Mir138-2*	microRNA 138-2	ENSMUSG00000065512	1.8782513	0.003919552
	snoRNA	*SNORA17*	Small nucleolar RNA SNORA17	ENSMUSG00000089591	1.145009	0.040112432
	snoRNA	*SNORA17*	Small nucleolar RNA SNORA17	ENSMUSG00000088425	4.6787663	0.000695565
	snoRNA	*SNORD86*	Small nucleolar RNA SNORD86	ENSMUSG00000088909	1.7270977	0.001484277
	misc_RNA	*Metazoa_SRP*	Metazoan signal recognition particle RNA	ENSMUSG00000092721	1.9833554	0.047319174
	misc_RNA	*7SK*	7SK RNA	ENSMUSG00000084547	2.1876316	0.023481824
	misc_RNA	*7SK*	7SK RNA	ENSMUSG00000087965	1.6663129	0.015273781
	snRNA	*U6*	U6 spliceosomal RNA	ENSMUSG00000094489	1.443963	0.021287415
	snRNA	*U6*	U6 spliceosomal RNA	ENSMUSG00000088933	3.7603142	5.8664E-05
	rRNA	*5S_rRNA*	5S ribosomal RNA	ENSMUSG00000088384	3.541263	0.001146871
	sense overlapping	*Gm20644*	predicted gene 20644	ENSMUSG00000093673	1.0921067	0.006876831
Downregulated	lincRNA	*Gm13025*	predicted gene 13025	ENSMUSG00000086159	-1.0466666	0.04115335
	lincRNA	*AC110567.1*	-	ENSMUSG00000097368	-1.3065866	0.000250179
	lincRNA	*AI480526*	expressed sequence AI480526	ENSMUSG00000090086	-2.4544034	0.003316961
	lincRNA	*Gm15567*	predicted gene 15567	ENSMUSG00000084974	-1.0093324	0.001714023
	lincRNA	*4933406I18Rik*	RIKEN cDNA 4933406I18 gene	ENSMUSG00000087475	-1.4427004	0.000988401
	lincRNA	*AC113484.1*	-	ENSMUSG00000096938	-2.069057	0.005108806
	lincRNA	*AC131780.3*	-	ENSMUSG00000097312	-1.8341787	0.002462086
	lincRNA	*4930520O04Rik*	RIKEN cDNA 4930520O04 gene	ENSMUSG00000074039	-1.2448175	0.012427459
	lincRNA	*Gm11946*	predicted gene 11946	ENSMUSG00000087291	-1.4263265	0.01559487
	lincRNA	*4933405E24Rik*	RIKEN cDNA 4933405E24 gene	ENSMUSG00000086209	-1.6460853	0.03286364
	lincRNA	*1700121N20Rik*	RIKEN cDNA 1700121N20 gene	ENSMUSG00000092066	-2.7382588	0.025925819
	lincRNA	*AC173345.2*	-	ENSMUSG00000097849	-1.856952	0.008906752
	lincRNA	*AC209577.1*	-	ENSMUSG00000097576	-1.1437159	0.040588282
	lincRNA	*AC154449.1*	-	ENSMUSG00000097768	-1.8573279	0.001016078
	lincRNA	*AC154507.1*	-	ENSMUSG00000097624	-2.2740648	0.010717467
	lincRNA	*AC145591.1*	-	ENSMUSG00000097755	-1.2007959	0.03728475
	lincRNA	*AC132389.1*	-	ENSMUSG00000097930	-2.0905662	0.001447383
	antisense	*Gm13362*	predicted gene 13362	ENSMUSG00000086418	-1.4526129	0.02259383
	antisense	*Gm13619*	predicted gene 13619	ENSMUSG00000052865	-2.0839705	0.002798275

antisense	*Pax6os1*	Pax6 opposite strand transcript 1	ENSMUSG00000086029	-1.3452741	0.039596412
antisense	*Gm13165*	predicted gene 13165	ENSMUSG00000086654	-1.5925248	0.02910456
antisense	*Gm15792*	predicted gene 15792	ENSMUSG00000085606	-2.435628	0.00059366
antisense	*RP23-271G7.3*	-	ENSMUSG00000097484	-1.0683532	0.04313622
antisense	*4933424L21Rik*	RIKEN cDNA 4933424L21 gene	ENSMUSG00000085719	-1.4564387	0.044925485
antisense	*C78197*	expressed sequence C78197	ENSMUSG00000087242	-3.0287738	0.037046853
antisense	*Gm11725*	predicted gene 11725	ENSMUSG00000085434	-1.03535	0.001938904
antisense	*C87198*	expressed sequence C87198	ENSMUSG00000092515	-1.4548206	0.000460192
antisense	*1110046J04Rik*	RIKEN cDNA 1110046J04 gene	ENSMUSG00000085457	-3.2520156	0.000140369
antisense	*Gm17116*	predicted gene 17116	ENSMUSG00000090785	-1.1741812	0.004966831
antisense	*Gm16191*	predicted gene 16191	ENSMUSG00000087693	-1.2607583	0.013894445
antisense	*Gm16194*	predicted gene 16194	ENSMUSG00000084956	-1.5119123	0.001481907
antisense	*Gm17080*	predicted gene 17080	ENSMUSG00000067389	-1.5519352	0.008406733
miRNA	*Mir291a*	microRNA 291a	ENSMUSG00000078008	-1.7085884	0.002841814
miRNA	*Mir291b*	microRNA 291b	ENSMUSG00000078032	-1.4041032	0.009981588
miRNA	*Mir293*	microRNA 293	ENSMUSG00000078035	-1.043071	0.018788872
miRNA	*Mir294*	microRNA 294	ENSMUSG00000077903	-1.153563	0.034180004
snoRNA	*SNORA29*	Small nucleolar RNA SNORA29	ENSMUSG00000065105	-1.064001	0.003222728
misc RNA	*7SK*	7SK RNA	ENSMUSG00000089475	-1.0603285	0.003452227
misc RNA	*Metazoa_SRP*	Metazoan signal recognition particle RNA	ENSMUSG00000093369	-1.2043077	0.018946713
snRNA	*U6*	U6 spliceosomal RNA	ENSMUSG00000064919	-1.5618181	0.000779107
snRNA	*U6*	U6 spliceosomal RNA	ENSMUSG00000089080	-1.0309738	0.002153195
non coding	*Sox2ot*	SOX2 overlapping transcript (non-protein coding)	ENSMUSG00000090828	-1.9579431	0.002018809
sense overlapping	*Gm20712*	predicted gene 20712	ENSMUSG00000093677	-1.2783619	0.007524139

Supplementary Table S1. Table listing all non-coding RNA entities with statistically significantly changes in nup155$^{+/-}$ embryonic stem cells compared to wild type controls. Pseudogenes and processed transcripts were omitted for clarity. Listed are the Gene Type, Gene Symbol, Gene Description from Mouse GENCODE database v17, Ensembl ID, Fold Change (FC), and corrected p value (p Corr) for each entity. Dashes denote entities which did not have a description at time of accession. Pseudogenes and processed transcripts were omitted for clarity.

Supplementary Table S2: Antibodies used in western blot and immunocytochemistry studies.

	Antibody	Host Species	Working Dilution	RRID #	Catalog Number	Manufacturer
**Primary**	SOX2	Goat	1:200 (WB)	AB_355110	AF2018	R&D systems (Minneapolis, MN)
	SOX2	Rabbit	1:100 (ICC)	AB_10585428	ab92494	Abcam (Cambridge, UK)
	NANOG	Rabbit	1:1000 (WB)<br>1:200 (ICC)	AB_386109	A300-398A	Bethyl Laboratories Inc. (Montgomery, TX)
	OCT4	Rabbit	1:150 (WB)<br>1:200 (ICC)	AB_445175	ab19857	Abcam (Cambridge, UK)
	NUP155	Rabbit	1:1000 (WB)	*	NBP2-22286	Novus Biologicals (Centennial, CO)
	β-TUBULIN	Rabbit	1:1000	AB_2210370	ab6046	Abcam (Cambridge, UK)
	LAMIN B1	Chicken	1:250	AB_10674021	ab90169	Abcam (Cambridge, UK)
**Secondary**	HRP	Goat	1:8000	AB_2313567	111-035-003	Jackson ImmunoResearch (West Grove, PA)
	HRP	Rabbit	1:8000	AB_2796231	6160-05	SouthernBiotech (Birmingham, AL)
	Alexa Fluor 594	Goat	1:500	AB_2534095	A11037	Fisher/Invitrogen (Waltham, MA)
	Alexa Fluor 488	Goat	1:500	AB_2633280	A32731	Fisher/Invitrogen (Waltham, MA)

Supplementary Table S2: Table listing details of antibodies used in western blot and immunocytochemistry experiments. Working dilutions used were from manufacturer concentrations, diluted with 1% non-fat powered milk in TBST. Resource Identification Initiative number (RRID #) was also included, extracted from the antibody registry database (http://antibodyregistry.org/). *Did not have an RRID#, catalog number is provided for anti-NUP155 (Novus Biologicals). HRP= Horseradish peroxidase, WB= West Blot, ICC= Fluorescent Immunocytochemistry.
